# The Teeth of the Rising Generation

**Published:** 1882-02

**Authors:** C. Edmund Kells

**Affiliations:** New Orleans, La.


### ARTICLE III.

### *The Teeth of the Rising Generation.*

BY C. EDMUND KELLS, JR., D. D. S., NEW ORLEANS, LA.

The subject which I present to you for your consideration this evening, is one that, to my mind, is fraught with no little interest to the dental profession of to-day, and appears to receive no attention from the able writers that are to be found within its ranks. To me it is a subject of never-ceasing anxiety.

How often are the little sufferers brought in to us, with teeth not yet fully erupted requiring our care in the arrest of caries. For such to be the case clearly proves something radically wrong, and when the cause is discovered a remedy for the evil must follow.

Allow the temporary set to first occupy our attention. When the child of four or five years of age is brought to me and placed under my care, the parent remarking that sleeplessness has reigned in the household for several nights past owing to one or more ashing teeth belonging to this diminutive specimen of humanity, I declare to you, gentlemen, in all secrecy of this occasion, my heart sinks within me, for I see "breakers ahead."

What to do, and how to do it, become the questions. An examination reveals, perhaps, one or two small crown cavities in the second molars, and the lower molars, carious upon their contiguous approximal surfaces—these latter mentioned being the cause of the trouble. The first mentioned, the crown cavities, I can say, I will probably manage to fill, and fill well, by which I mean, put in amalgam (never gold) and save that portion of the tooth from further inroads of decay for the remainder of its time. But when it comes to the approximal cavities, I must admit, I can in the majority of cases do nothing to my entire satisfaction, and regret the same exceedingly. In the first place, the enamel of the temporary teeth is of such structure as to decay about as rapidly, if not more so, than the dentine beneath, the resulting cavity being saucer-shaped and practically no place for undercuts to retain a filling, owing to the close approximation of the pulp. Again, when these cavities extend to the margin of the gum—which abtains not infrequently—to keep them dry is a feat in itself to perform. So here I am often forced to use a cement filling (agate I prefer) on account of the ease and rapidity with which it may be inserted, and when necessary refill as before, which may be required several times during the life of the teeth, my aim being to keep them in place until the proper time for their loss; but if that be impossible maintain them at all hazards (where such a thing is possible) until the six-year molars take their places in the rear, and then, if the worst comes, out must come the offending tooth. This is my practice. If any man can say he fills all temporary teeth in a perfect manner and saves them, I say, " Friend, give me your hand ;" and also add, " But let me see how you do it."

That the teeth of the rising generation are far, very far, below the standard of those of their parents and more remote progenitors, is a conclusion that has been forced upon my mind from practical observation. Who among us has not little patients from twelve to fourteen years of age, under his care, with ten or fifteen, yes, and even twenty, monuments of his dental skill in their mouths? Should such things be? And how often does a mother say: " This passes my understanding. My daughter takes the best of care of her teeth, and yet she has already had more work done than I!" And such a statement proves true. Cases obtain in which better care could not be taken of the dental arch, and still every year we find one or more carious spots appearing. However, in the class cited we find that goad care is taken and pride enters as a factor therein; and so it is not quite so bad as it might be. That is one picture, not a bright one for the patient (however it may be for the operator,) to be sure, but with proper constitutional treatment and the regimen looked after we may hope for an improvement.

Here is another: Hardly a year has passed since this little patient left your hands, teeth in A No. 1 condition, a number of fillings having been inserted—contemplated with pride. What do you find to-day? Tartar, collections of food about the teeth, cavities. Yes, all these do you find—thanks to a regular habit of abstaining from the use of brush or anything else of cleanly tendencies! What a picture for a conscientious man to look upon! Among a number of cavities here found is it any wonder that one or two of the a year-ago admired fillings have failed? Or rather, is it not a wonder that any have stood? This is certainly a deplorable outlook, for do you not see failure of your best endeavors stare you in the face?

Such are examples of cases which I fear we all find but too frequently in our practice.

What is to be done?

That decay is but a breaking up of tooth structure due to chemical action is to my mind the true theory, the Bacteria-men to the contrary, notwithstanding. Therefore the reduction of the secretions of the mouth to their normal conditions must be accomplished, when found necessary, during the same time that other required work is being performed. And what material is to be used in filling the teeth, especially those of the anterior portion of the arch and which are sure to contain large approximal cavities with thin walls, and the teeth themselves of a very soft texture? Do you say gold? Though that is what I would like to say, I am compelled to admit I do not always use it at this stage of the case, for the simple reason of being afraid to do so. Soft tooth, bad habits, give poor prospects, say I. So, then I consider it advisable to fill these with a temporary filling of agate cement, which is to remain a year or so, and also endeavor during that period to instill into the mind of the patient some notion of pride about his appearance; and also trace them (the teeth) up through constitutional treatment. In any case in which the nerve is nearly or quite exposed a thin layer of os-artificiel mixed with creosote is placed over it. At the end of the required time refill with gold, and then I expect as a reward for your trouble the greater permanency of your work.

Though by no means a "new departurist," I am just as far from being a "nothing-but-gold" practice man. But rather believing that no one material is best for filling teeth, do I take into consideration the facts governing each case, and then decide. With a decided preference for gold, I am often found deciding in favor of amalgam in many of these cases where the posterior teeth are involved and the structure is of a soft nature, and in all cases in which the cavities are, so to speak, inaccessible. In all cases should the contour be restored, particularly upon the approximal surfaces. In separating, I do not believe; for, run a file or a disk between two molars to-day, and a year hence you may expect to find those teeth together again and in worse condition than before your operation.

Most strenuously must be urged the necessity of keeping the teeth absolutely free from all particles of food by the careful use of brush, and pick, and silk or rubber. The style of brush must be insisted upon—small, short bristles, not too hard, and concaved longitudinally in shape. And above all, the proper manner of using the brush—which I confidently believe will prove a novelty to twenty-four out of every twenty-five patients to whom you advise it—must be particularly dwelt upon. Recession of the gums and furrowing at the necks of the teeth is not an uncommon occurrence, due to a hard brush, and lateral use of the same upon the buccal and labial surfaces. Why such use of the brush must never be made may be clearly demonstrated, and then, and then only, can it be hoped to make any impression upon the mind of the patient who prides himself upon the thorough manner of performing that operation.

Neither metal nor wood picks should be used—quill being the best. In all cases should silk or something to that effect be passed regularly between the teeth, for in no other manner can all the debris of food be removed. The unwinding, cutting off and waxing required by floss silk being somewhat troublesome, this duty is often neglected, from that cause alone; and to obviate this difficulty, and to render the means as ready as possible, I frequently advise the use of small rubber rings in the place of silk, and which answers the purpose fully as well. No. 8 is the mark by which the suitable size is designed by the stationer, from whom they may be obtained. These I find very popular, being decidedly superior to the floss in the matter of convenience, being best kept of ready access, and a few carried in the pocket or purse.

The use of a suitable mouth wash after the taking of any remedy having an injurious effect upon the teeth, should also be urged, and sorry am I to have observed the little attention paid to this by the medical men who prescribe such remedies with never a thought for the teeth, important factors as they are in the general health of the subject.

But here the end is not yet. Though the dental organs have been restored to as near a perfect condition as possible, and the oral fluids are again in a normal state, and all this advice beaten into their heads with the full force of the mallet—this is not enough. Constitutional treatment must follow, and the diet receive proper attention. Phosphorous and lime are the lacking elements, and they must be supplied. The syrup of lacto-phosphate of lime is the rock upon which I build my hopes. A teaspoonful three times daily, during alternate weeks, should be regularly taken until a decided change for the better is observed. Unbolted flour in its various forms should take the place of the fine white flour used, and the latter prohibited from the table. Oat meal, buckwheat, graham crackers, bread or muffins, and lean beef must be depended upon to build up these tissues.

In view of the general tendency towards defective teeth, and considering the fact that during the process of calcification is the period when they are in greatest need of a full supply of nutritive material, I can but think that the greatest good will obtain by the exhibition of the syrup to the mother of the unborn child, and afterwards continued *in propria persona.*

You will notice I have only cited the class of teeth of poor organization, and the treatment therefor. No reference is made to those of a dense hard quality, with little or no tendency to decay.

Only to the most persistent efforts, directed in every exposed quarter, may we expect any reasonable amount of success in the class of cases to which I have called attention.—*Dent. Luminary.*

